# Genetic Polymorphism of NOS3 with Susceptibility to Deep Vein Thrombosis after Orthopedic Surgery: A Case-Control Study in Chinese Han Population

**DOI:** 10.1371/journal.pone.0070033

**Published:** 2013-07-26

**Authors:** Jizheng Qin, Jin Dai, Zhihong Xu, Dongyang Chen, Jianghui Qin, Dongquan Shi, Huajian Teng, Qing Jiang

**Affiliations:** 1 The Center of Diagnosis and Treatment for Joint Disease, Drum Tower Hospital Affiliated to Medical School of Nanjing University, Jiangsu, PR China; 2 Joint Research Center for Bone and Joint Disease, Model Animal Research Center (MARC), Nanjing University, Jiangsu, PR China; Yale School of Public Health, United States of America

## Abstract

Deep vein thrombosis is one of the common complications of orthopedic surgery. Studies indicated that genetic factors played a considerable role in the pathogenesis of deep vein thrombosis. Endothelial nitric oxide synthase which encoded by nitric oxide synthase 3 (NOS3), can generate nitric oxide in endothelial cells. As a predominant regulator for vascular homeostasis, nitric oxide might be involved in the pathogenesis of thrombosis. It had been proved that the NOS3 polymorphism (rs1799983) was associated with the development of cardiovascular diseases. Our objective was to evaluate the association between the NOS3 polymorphism (rs1799983) and deep vein thrombosis after orthopedic surgery in Chinese Han population. The polymorphism was genotyped in 224 subjects with deep vein thrombosis after orthopedic surgery and 580 controls. Allele and genotype frequencies were compared between subjects with deep vein thrombosis and control subjects. The allele and genotype frequencies of the NOS3 polymorphism (rs1799983) were significantly different between subjects with deep vein thrombosis and control subjects. There were also significant differences when the subjects were stratified by gender, surgery type and hypertension status. These findings suggested that the NOS3 polymorphism (rs1799983) was associated with susceptibility to the deep vein thrombosis after orthopedic surgery in Chinese Han population, and NOS3 might play a role in the development of deep vein thrombosis after orthopedic surgery.

## Introduction

Deep vein thrombosis (DVT) is one of the common complications of orthopedic surgery. It usually occurs at the deep veins of legs (such as the calf vein, femoral vein, and popliteal vein) or the deep veins of pelvises after total knee arthroplasty (TKA) and total hip arthroplasty (THA). The incidence of DVT is up to 50–60% in patients after arthroplasty [1], and pulmonary embolism (PE) which is one of its lethiferous complications can lead to severe mortality. According to the report from American Heart Association, approximately 2,000,000 people suffered from DVT per year and 10% of these died from PE, in the United States alone [2]. Results of some genetic studies had shown that genetic factors might contribute to the development of DVT [3], [4].

Nitric oxide synthase 3 (NOS3) locates at chromosome 7q36, and it encodes endothelial nitric oxide synthase (eNOS), which can generate nitric oxide (NO) in endothelial cells. Endothelial NO is a key determinant of vascular homeostasis. It can regulate several physiological processes, including platelet adhesion, leukocyte adhesion, endothelial cell migration and vascular smooth muscle cell migration. And it can also participate in vascular repair. Dysfunction of any of these processes may result in atherosclerotic and thrombotic diseases [5]–[8]. Therefore, it is conceivable that the NOS3 mutations may be involved in the development of DVT by affecting the generation of NO in endothelial cells and vascular homeostasis. The previous studies had shown the association of a single nucleotide polymorphism (SNP) in the NOS3 gene (rs1799983) with the risk of coronary artery disease and venous thromboembolism (VTE) [9]–[13], and two other polymorphisms of NOS3 (2786 T/C and intron4, 27 bp repeat) were associated with the susceptibility to VTE in the Caucasian population [14]. Moreover, deep vein trauma is a common complication in orthopedic surgery, and it is obvious that deep vein trauma is a risk factor of DVT [1]. Impaired NO bioavailability might reduce the ability of vascular repair [5]–[8], which might increase the risk of DVT after deep vein trauma.

Together, these studies implicated the candidacy of NOS3 as a susceptibility gene for DVT after orthopedic surgery. However, there was no report of association between NOS3 and DVT after orthopedic surgery. The objective of this research was to appraise the genetic association between the polymorphism (rs1799983) in NOS3 gene and DVT after orthopedic surgery in Chinese Han population.

## Design and Methods

### 2.1. Ethics Statement

Our research was conducted in accordance with the Declaration of Helsinki. This study was approved by the ethics review committee of the Drum Tower Hospital affiliated to Medical School of Nanjing University, and all participants provided their written informed consent to participate in this study.

### 2.2. Subjects

804 consecutive eligible subjects underwent orthopedic surgery on knee or hip were recruited at the Center of Diagnosis and Treatment for Joint Disease, Drum Tower Hospital affiliated to Medical School of Nanjing University. Of these 804 subjects, 224 cases suffered from DVT after orthopedic surgery (161 females and 63 males) and 580 controls showed no evidence of DVT (349 females and 231 males). For each subject, DVT was assessed using venography, only subjects with filling defect of venous lumen were classified as cases, subjects without filling defect of venous lumen were classified as controls, and the healthy controls had no history of VTE or clinical evidence of VTE. Hypertension was defined as systolic blood pressure >140 mmHg and/or diastolic blood pressure >90 mmHg on an average of two measurements or by current anti-hypertensive treatment by WHO (World Health Organization) criteria. All subjects enrolled in this study were Han Chinese origin living in or around Nanjing. No subject withdrew from this study.

### 2.3. Venography

DVT was evaluated by performing venography within 7 days postoperatively for all these 804 subjects. The following venous segments were observed: common femoral veins, superficial femoral veins, anterior and posterior tibial veins, peroneal veins, popliteal veins. The criterion for diagnosis of DVT was a filling defect of venous lumen in two or more views according to the American College of Chest Physicians Evidence-Based Clinical Practice Guidelines of diagnosis of DVT [15].

### 2.4. Genotyping Analysis

The NucleoSpin Blood QuickPure Kit (Macherey-Nagel GmbH & Co. KG, Düren, German) was used to extract DNA from peripheral blood following the manufacturer’s protocol. The SNP rs1799983 was genotyped by using Taqman assay (Applied Biosystems 7500, ABI, Foster City, CA, USA), according to the manufacturer’s instruction.

### 2.5. Statistical Analysis

The previous studies had shown that the G allele played a protective role in coronary artery disease and VTE [9]–[13], thus, we proposed that GT and TT genotypes might be the risk genotypes in DVT after orthopedic surgery, and the dominant genetic model was used in our study, however, we also calculated the TT vs. other combined to find whether or not the recessive genetic model might exist in our study. Chi-squared test was used to analyze the significances of allele frequency difference and genotypes distribution difference between subjects with DVT and control subjects. Hardy–Weinberg equilibrium was used to verify allele frequency and genotype distribution. Clinical variables gender, surgery type and hypertension status of all subjects with different genotype were compared using the chi-square test. Odds ratios (ORs) and 95% confidence intervals (CIs) were calculated by use of multivariate logistic regression analyses. A *p* value of <0.05 was considered statistically significant in this study. These tests were implemented by using SPSS 12.0 system software (SPSS Inc., Chicago, Illinois, USA).

### Results

The basic characteristics of the study subjects were summarized in Table 1. The cases and controls were matched by age, BMI, ethnicity origin, residence area, average operation times, hypertensive status, surgery type and type of anesthetics (all accepted general anesthesia), individually (all *p*>0.05). The Hardy–Weinberg equilibrium of all subjects was tested which indicated there was not genotyping error in our dataset (*p*  = 0.806). The allele and genotype frequencies of DVT subjects and control subjects were shown in Table 2. The frequencies of GG, GT, TT genotypes for the SNP rs1799983 in DVT subjects were 72.8%, 25.4% and 1.8%, respectively, meanwhile in control subjects were 81.0%, 17.8% and 1.2%, respectively. The genotype distributions were conformed to Hardy-Weinberg equilibrium in both cases and controls (*p*  = 0.70 and 0.61, respectively) (Table 2). Significant differences were detected in the comparison of allele frequency (*p*  = 0.012) and in the comparison of GG genotype versus other genotypes combined (*p*  = 0.010) between cases and controls (Table 3). The significant differences still existed when adjusted by age, gender and BMI. No significant difference was observed in the comparison of TT genotype versus other genotypes combined (*p*  = 0.5265) (Table 3).

**Table 1:** Subject characteristics.

Subjects	DVT cases	Controls	*p* value
**Age**	60.2±10.5	58.6±10.1	0.059
**Sex**			
** male**	63(28.2)	231(39.8)	0.002*
** female**	161(71.8)	349(60.2)	
**BMI**	26.1	25.9	0.396
**Surgery type**			
** hip**	125	333	0.679
** knee**	99	247	
**Hypertensive status**			
** hypertensive**	63	152	0.582
** normotensive**	161	428	

* *p* was considered statistically significant.

**Table 2:** Genotype and allele frequencies of G/T transition SNP (rs1799983) of the NOS3 gene in Han Chinese population.

Subjects	Number	Allele		Genotype			Hardy–Weinbergequilibrium *p* value
		G (%)	T (%)	GG (%)	GT (%)	TT (%)	
**All cases**	224	383 (85.5)	65 (14.5)	163 (72.8)	57 (25.4)	4 (1.8)	0.6999
**All controls**	580	1043 (89.9)	117 (10.1)	470 (81.0)	103 (17.8)	7 (1.2)	0.6146
**Female cases**	161	265 (82.3)	57 (17.7)	106 (65.8)	53 (32.9)	2 (1.3)	
**Female controls**	349	628 (90.0)	70 (10.0)	283 (81.1)	62 (17.8)	4 (1.1)	
**Male cases**	63	118 (93.7)	8 (6.3)	57 (90.5)	4 (6.3)	2 (3.2)	
**Male controls**	231	415 (89.8)	47 (10.2)	187 (90.0)	41 (17.7)	3 (1.3)	
**Hip cases**	125	216 (86.4)	34 (13.6)	93 (74.4)	31 (24.8)	1 (0.08)	
**Hip controls**	333	607 (91.1)	59 (8.9)	277 (83.2)	51 (15.3)	5 (1.5)	
**Knee cases**	99	166 (83.8)	32 (16.2)	70 (70.7)	26 (26.3)	3 (3.0)	
**Knee controls**	247	438 (88.7)	56 (11.3)	193 (78.1)	52 (21.1)	2 (0.8)	
**Hypertensive cases**	63	108 (87.5)	18 (12.5)	45 (71.4)	18 (28.6)	0 (0.0)	
**Hypertensive controls**	152	289 (95.1)	15 (4.9)	137 (90.1)	15 (9.9)	0 (0.0)	
**Normotensive cases**	161	275 (85.4)	47 (14.6)	118 (73.3)	39 (24.2)	4 (2.5)	
**Normotensive controls**	428	754 (88.1)	102 (11.9)	333 (77.8)	88 (20.6)	7 (1.6)	

* *p* was considered statistically significant.

**Table 3:** Association of the NOS3 polymorphism (rs1799983) with DVT when stratified by gender.

Groups compared	GG vs. other combined			TT vs. other combined			G allele vs. T allele			All genotype
	OR	*p* value*	95% CI	OR	*p* value*	95% CI	OR	*p* value*	95% CI	*p* value*
**All cases vs. all controls**	1.60	0.010*	1.12 to 2.29	1.49	0.526	0.43 to 5.13	0.66	0.012*	0.48 to 0.91	0.037*
**All cases vs. all controls** **adjusted by age, sex and BMI**	1.58	0.015*	1.09 to 2.27	1.61	0.457	0.46 to 5.63	0.66	0.016*	0.48 to 0.92	0.019*
**Female cases vs. female controls**	2.22	0.000*	1.46 to 3.39	1.08	0.926	0.20 to 5.99	0.52	0.001*	0.36 to 0.76	0.001*
**Female cases vs. female controls** **adjusted by age and BMI**	2.36	0.000 *	1.54 to 3.63	1.17	0.859	0.21 to 6.51	0.49	0.000 *	0.34 to 0.72	0.000*
**Male cases vs. male controls**	0.45	0.074	0.18 to 1.10	2.49	0.307	0.41 to 15.25	1.67	0.191	0.77 to 3.63	0.056
**Male cases vs. male controls** **adjusted by age and BMI**	0.44	0.079	0.18 to 1.10	2.47	0.332	0.40 to 15.42	1.68	0.189	0.77 to 3.67	0.036*

* *p* was considered statistically significant.

Association between gender and genotype distribution of rs1799983 were also performed in prior study [12], to verify the gender specific association between the SNP rs1799983 and DVT, we compared frequencies of the allele and genotype after stratification by gender. In female subjects, significant differences were observed in the comparison of allele frequency (*p*  = 0.001) (Table 3) and in the comparison of GG genotype versus other genotypes combined (*p*  = 0.000) (Table 3), but no significant difference was observed in the comparison of TT genotype versus other genotypes combined (*p*  = 0.926) (Table 3). The significant differences still existed when adjusted by age, and BMI. In male subjects, no significant difference was found in any comparison (all *p*>0.05) (Table 3).

Significant differences were observed in the comparison of GG genotype versus other genotypes combined (*p*  = 0.034) (Table 4) in hip surgery group after stratification by surgery type, and no significant difference was found in any comparison (all *p*>0.05) (Table 4) in knee surgery group. However, no significant differences were observed in hip surgery group when adjusted by age, gender and BMI, in contrast, significant differences were observed in the comparison of allele frequency (*p*  = 0.043) (Table 4) in knee surgery group when adjusted by age, gender and BMI.

**Table 4:** Association of the NOS3 polymorphism (rs1799983) with DVT when stratified by surgery type.

Groups compared	GG vs. other combined			TT vs. other combined			G allele vs. T allele			All genotype
	OR	*p* value*	95% CI	OR	*p* value*	95% CI	OR	*p* value*	95% CI	*p* value*
**Cases vs. controls in hip surgery group**	1.70	0.034*	1.04 to 2.79	0.53	0.556	0.06 to 4.57	1.51	0.073	0.96 to 2.37	0.056
**Cases vs. controls in hip surgery group** **adjusted by age, sex and BMI**	1.35	0.268	0.80 to 2.28	0.54	0.583	0.06 to 4.80	1.22	0.400	0.77 to 1.95	0.209
**Cases vs. controls in knee surgery group**	1.48	0.143	0.87 to 2.51	3.83	0.118	0.63 to 23.29	1.51	0.085	0.94 to 2.41	0.151
**Cases vs. controls in knee surgery group** **adjusted by age, sex and BMI**	1.64	0.079	0.94 to 2.84	4.02	0.136	0.64 to 25.03	1.62	0.048 *	1.00 to 2.63	0.139

* *p* was considered statistically significant.

Moreover, significant differences were observed in the comparison of allele frequency (*p*  = 0.001) (Table 5) and in the comparison of GG genotype versus other genotypes combined (*p*  = 0.001) in the hypertensive group after stratification by hypertension status. The significant differences still existed when adjusted by age, gender and BMI. No significant difference was observed in the normotensive group.

**Table 5:** Association of the NOS3 polymorphism (rs1799983) with DVT when stratified by hypertension status.

Groups compared	GG vs. other combined			TT vs. other combined			G allele vs. T allele			All genotype
	OR	*p* value*	95% CI	OR	*p* value*	95% CI	OR	*p* value*	95% CI	*p* value*
**Hypertensive cases vs. hypertensive controls**	3.65	0.001*	1.70 to 7.84	–	–	–	3.21	0.001*	1.56 to 6.60	0.001*
**Hypertensive cases vs. hypertensive controls** **adjusted by age, sex and BMI**	4.50	0.000*	1.95 to 10.42	–	–	–	3.50	0.001*	1.65 to 7.44	0.000*
**Normotensive cases vs. normotensive controls**	1.28	0.249	0.84 to 1.94	1.53	0.498	0.44 to 5.31	1.26	0.217	0.87 to 1.83	0.475
**Normotensive cases vs. normotensive controls** **adjusted by age, sex and BMI**	1.31	0.215	0.86 to 2.01	1.74	0.394	0.49 to 6.20	1.30	0.175	0.89 to 1.90	0.286

* *p* was considered statistically significant.

## Discussion

This study was the first attempt to evaluate the association of the NOS3 polymorphism (rs1799983) with the risk of DVT after orthopedic surgery. On the basis of this study, we demonstrated that there were significant differences in genotype and allele frequencies between DVT subjects and control subjects, the GT+TT genotype and T allele frequencies were significantly higher in cases compared to controls, thus, we stated that the T allele might play a risk role in subjects after orthopedic surgery. To test whether or not the gender, surgery type and hypertension status could impact the results, we stratified the subjects by gender, surgery type and hypertension status, and statistical analyses were performed in these subgroups, we also observed that there were significant differences in genotype and allele frequencies between the cases and controls when Bonferroni’s correction was applied. Our findings were similar to some previous studies which had shown the association of the single nucleotide polymorphism (SNP) in the NOS3 gene (rs1799983) with the risk of coronary artery disease and venous thromboembolism (VTE) [9]–[13]. In these prior study, patients with T allele of rs1799983 was prone to suffer from VTE, which was line up with the results of our study. Prior literature about the association between VTE and rs1799983 in Chinese population shown that the frequency of GT+TT genotype was significantly higher in VTE patients compared to controls (20.3% vs. 13.4%), and T allele carriers were significantly higher in VTE subjects compared to the controls (11.3% vs. 7.3%) [12], the same trend were observed in our study (GT+TT genotype: 27.2% vs. 19.0%, T allele: 14.5% vs. 10.1%).

Stratification of subjects by gender revealed that significant differences in genotype and allele frequencies between the cases and controls in female subjects, the GT+TT genotype and T allele frequencies were also significantly higher in female cases compared to female controls, it was suggested that the G allele might play a protective role against T allele in female subjects after orthopedic surgery. Whereas, no significant difference was observed in male subjects, and the OR values in the comparison of allele frequency and in the comparison of GG genotype versus other genotypes combined were controversial to those in the comparisons in female subjects. This SNP might be not associated with DVT after orthopedic surgery in male subjects. Association between gender and genotype distribution of rs1799983 were also performed in prior study, the significant differences were observed in male subgroup, and no significant differences were observed in female subgroup [12], which was adverse to our study, one explanation for this opposition was the proportions of men and women in the case and control groups were different, more cases were observed in female group and more controls were observed in male group in our study, as the sample size of male subjects was small, further research with more samples would be necessary to deny the association.

Significant differences were observed in the knee surgery group, but no significant differences were observed in the hip surgery group when stratified by surgery type and adjusted by age, gender and BMI. The T allele frequencies were significantly higher in cases compared to controls in the knee surgery group, which suggested that subjects with T allele was prone to DVT after knee surgery. The OR values in the comparison of allele frequency and in the comparison of GG genotype versus other genotypes combined were similar between the two groups, it indicated that the trends of allele frequency and genotype distribution were consistent between the two groups. The explanation of negative results in knee surgery group was the relatively small sample size, which might limit the statistical power. To check the possible association, further researches should be implemented.

Moreover, significant association was observed in the hypertensive group, and no significant association was observed in the normotensive group when stratified by hypertension status. The GT+TT genotype and T allele frequencies were significantly higher in cases compared to controls in the hypertensive group. These results indicated that hypertensive patients with T allele were prone to DVT after orthopedic surgery, and the SNP rs1799983 might be associated with DVT after orthopedic surgery in hypertensive patients. Compared to the normotensive subjects, NOS3 might make more contribution in the etiology of DVT after orthopedic surgery in hypertensive subjects. However, we cannot calculate the *p* value the comparison of TT genotype versus other genotypes combined in the hypertensive group and the *p* value of all genotypes. The explanation for this discrepancy was the small size of the subjects. Thus, we could not make a definite conclusion because the sample size was small. Further studies with more samples would be necessary to prove the association.

In NOS3, rs1799983 locates at c.894 of the coding region, and c.894G/T corresponds to glutamic acid and aspartic acid at position 298 of eNOS protein, respectively. eNOS protein with aspartic acid was unstable compared to that with glutamic acid [16]. As a result, the T allele may result in reduced function of the eNOS protein, then, lead to the decrease of NO synthesis in endothelial cells, and it would finally increase the risk of atherosclerotic and thrombotic diseases including DVT after orthopedic surgery. In humans, the association of endothelial dysfunction with hypertension had been reported in the coronary and forearm vascular beds [17]–[19]. Hypertension could cause endothelial dysfunction by reducing NO synthesis in endothelial cells [20]–[22], and the abilities of repairing damaged vascular and inhibiting platelet aggregation and smooth muscle cell proliferation might be suppressed severely in hypertensive subjects, therefore, the hypertensive subjects were inclined to suffer from DVT after orthopedic surgery, and the mutation of NOS3 might play a more important role of DVT after orthopedic surgery in hypertensive subjects compared with normotensive subjects. These might explain the incidence of DVT in hypertensive group was higher than that in normotensive group, and significant differences were only observed in hypertensive group (Table 5).

In conclusion, the present study demonstrated that there might be a significant association between the NOS3 polymorphism (rs1799983) and susceptibility to DVT after orthopedic surgery in Chinese Han population. Further studies would be needed to be conducted to confirm the association of this polymorphism with DVT after orthopedic surgery in Chinese Han population and in other populations.
